# Molecular detection and characterization of *Shigella* spp. harboring extended-spectrum β-lactamase genes in children with diarrhea in northwest Iran

**DOI:** 10.1186/s40348-022-00152-0

**Published:** 2022-12-08

**Authors:** Sahar Sabour, Amir Teimourpour, Jafar Mohammadshahi, Hadi Peeridogaheh, Roghayeh Teimourpour, Taher Azimi, Zahra Hosseinali

**Affiliations:** 1 Department of Microbiology, School of Medicine, Ahvaz Jundishapur University of Medical Sciences, Ahvaz, Iran; 2 Department of Microbiology, School of Medicine, Ardabil University of Medical Science, Ardabil, Iran; 3 Blood Transfusion Research Center, High Institute for Research and Education, Tehran, Iran; 4 Department of Infectious Diseases, School of Medicine, Ardabil University of Medical Sciences, Ardabil, Iran; 5 Zoonoses Research Center, Ardabil University of Medical Sciences, Ardabil, Iran; 6 Genomics Research Center, Ardabil University of Medical Sciences, Ardabil, Iran; 7 Department of Bacteriology & Virology, School of Medicine, Shiraz University of Medical Science, Shiraz, Iran

**Keywords:** *Shigella* spp., *Shigella sonnei*, Extended-spectrum β-lactamase, Diarrhea, Iran

## Abstract

Shigellosis is one of the acute bowel infections and remains a serious public health problem in resource-poor countries. The present study aimed to survey the distribution of extended-spectrum β-lactamase (*ESBL*)-producing *Shigella* strains isolated from patients with diarrhea in northwest Iran. In the present cross-sectional study, from January 2019 to December 2020, 1280 fecal samples were collected from children with diarrhea in Ardabil, Iran. Multiplex PCR assay was applied for the presence of *ipaH*, *invC*, *wbgZ*, *rfpB*, and *rfc* genes to detect *Shigella* spp., *Shigella sonnei*, *Shigella dysenteriae*, *Shigella flexneri*, and *Shigella boydii*, respectively. Phenotypic detection of ESBL-producing isolates was carried out using the Double Disc Test (DDT). The frequency of main ESBL encoding genes including *bla*_*CTX-M*_, *bla*_*SHV*_, and *bla*_*TEM*_ was detected using multiplex PCR. The genetic similarity of *S. sonnei* isolates was determined using ERIC PCR. A total of 49 *Shigella* isolates (3.8%; 49/1280) including 42 (85.7%) *S. sonnei*, 5 (10.2%) *S. flexneri*, and 2 (4%) *S. dysenteriae* were identified. *S. boydii* was not detected in any fecal samples. ESBLs were produced by 10.2% of *Shigella* spp. including 3 *S. sonnei*, 1 *S. flexneri*, and 1 *S. dysenteriae*. The ESBL encoding genes include *bla*_*CTX-M*_ and *bla*_*TEM*_ found in 65.3% and 61.2% of isolates, respectively. *bla*_*SHV*_ gene was not detected in any isolates. The ERIC-PCR profiles allowed the differentiation of 42 *S. sonnei* strains into 6 clusters. Our study revealed a high frequency of ESBL-encoding genes among *Shigella* spp. in northwest Iran. The high prevalence of *S. sonnei* harboring ESBL genes, in the present work, is the main challenge for dysentery treatment, and this concern justifies the need for effective and regular monitoring of antibiotic usage among patients.

## Introduction

Shigellosis is one of the main acute bowel diseases and remains a major public health problem in resource-poor countries. This disease is caused by gram-negative bacteria belonging to Enterobacteriaceae [1]. In general, *Shigella* species are facultative intracellular and non-flagellated clinically important pathogens. *Shigella flexneri* (*S. flexneri*) with 10 serotypes, *Shigella dysenteriae* (*S. dysenteriae*) with 12 serotypes, *Shigella boydii* (*S. boydii*) with 18 serotypes, and *Shigella sonnei* (*S. sonnei*) with 1 serotype are the *four major* serological groups of *Shigella* spp. [2, 3]. The infectious dose of *Shigella* species is very low (10 to 100 organisms), and the fecal-oral route represents the main transmission line to shigellosis [4]. According to several factors such as partially developed immunity*,* poor hygiene, and lack of past exposures, the age group less than 5 years is highly susceptible to *Shigella* infections*.* Among developing countries and in children aged < 5 years, diarrhea is the second most common cause of death [5]. The use of effective antibiotics for the treatment of *Shigella* infections may lead to a reduction in disease transmission and prevention of lethal outcomes [6]. However, despite the existence of effective treatment regimens, shigellosis continues to be a major global health challenge with estimated shigellosis deaths of 28,000 to 48,000 among young children in 2013 [7]. Antibiotic therapy is usually recommended for dealing with shigellosis because it may shorten the clinical course of the disease, reduce the risk of transmission, and prevent potentially fatal complications [8]. Among the different groups of antibiotics, β-lactams and quinolones are usually used against *Shigella* infections. However, over the past half-century, many countries have reported the high resistance of *Shigella* species to common antimicrobial agents [9]. Moreover, based on the geographical location, the antibiotic resistance profile of *Shigella* spp. varies, and the treatment process is difficult [6, 10].

β-Lactams are valuable drugs for treating various types of bacterial infectious diseases [11]. The excessive use of β-lactam antibiotics has led to an increase in resistance to them, especially in gram-negative bacteria. β-lactamases are enzymes that bacteria use to break the β-lactam ring of β-lactam antibiotics and become resistant to them [12, 13]. Different types of β-lactamases have been identified, which differ from each other in terms of structural characteristics and molecular targets, and amino acid sequences [14]. Accordance to Ambler’s classification, β-lactamases are divided into four groups including A, B, C, and D. Extended-spectrum β-lactamase (ESBL) is a group of β-lactamase that confer resistance to potent β-lactams such as third-generation cephalosporins [15]. ESBL-producing *Shigella* spp. has been identified as a major concern in hospital and community-acquired infections worldwide, especially in developing county [16]. In this regard, the present study aimed to survey the distribution of *ESBL*-producing *Shigella* strains isolated from patients with diarrhea in northwest, Iran.

## Materials and methods

### Study area, sample collection, and bacterial isolates

**Fig. 1:**
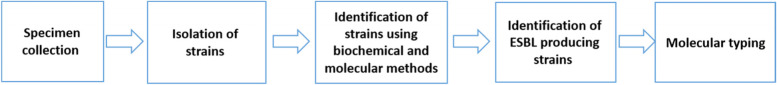
A flowchart of method steps

The present research was performed in Ardabil, an ancient city in northwestern Iran. From January 2019 to December 2020, 1280 fecal samples were collected from children with diarrhea who had been referred to the laboratory of Bu Ali Hospital at Ardabil University, belonging to the Iranian health system in Ardabil, Iran. The fecal samples were cultured on MacConkey agar and selenite F medium, and all plates were incubated overnight at 37 °C. After incubation time, all colonies were transferred to Hektoen enteric agar (HE) and xylose-*lysine deoxycholate* agar (*XLD* agar), (Merck, Hamburg, Germany) and were incubated at 37 °C for 18 to 24 h. Specimens with green colonies on HE medium, colorless colonies on XLD medium, and non-lactose fermenting colonies on MacConkey agar were suspected of *Shigella* species. The final identification of *Shigella* species was conducted using conventional biochemical tests such as *triple sugar iron* (*TSI*), *SIM* medium (sulfide indole motility medium), ornithine decarboxylase (ODC), lysine iron agar (LIA), Simmons citrate, and urea agar. In the next step, all confirmed bacteria were stored at −80 °C in 10% glycerol (Fig. 1).

### Molecular detection of Shigella species

DNA was extracted using the DNA extraction kit (Sinaclon, Tehran, Iran) according to the manufacturer’s instructions. The NanoDrop device (Thermo Fisher Scientific, Waltham, MA, USA) was used for the evaluation of the quality and quantity of the extracted DNA. Molecular identification of *Shigella* species was performed using multiplex PCR assay. The specific genes including *ipaH*, *invC*, *wbgZ*, *rfpB*, and *rfc* were employed to detect *Shigella* spp., *S. sonnei*, *S. dysenteriae*, *S. flexneri*, and *S. boydii*, respectively [17, 18]. The sequence results were submitted in GenBank (accession numbers: MN503255.1 and MN503256.1).

The sequence of the primers used for multiplex PCR assay is shown in Table 1. The PCR reaction was performed in a thermocycler (Bio-Rad, Germany) as follows: 1 cycle at 94 °C for 4 min, 32 cycles at 94 °C for 45 s, different annealing temperatures for each gene for 45 s, 72 °C for 40 s, and the final extension cycle at 72 °C for 10 min. The PCR reaction was carried out at the final volume of 25 μl including 12.5 μl of Master Mix, 0.5 μl of 10 pM forward and reverse primers, and 0.5–1 μl of genomic DNA. All the materials used in the PCR reaction were purchased from SinaClon BioScience Company, Iran. *S. flexneri* ATCC 12022 and *S. sonnei* ATCC 9290 were used as a positive control in the PCR reaction. Moreover, DNase-free as a negative control was used for PCR assay. The PCR products were stained by safe stain and screened by electrophoresis on 1–1.5% agarose. The bands associated with PCR products were observed through the application of a transilluminator.

**Table 1:** Primers used for the detection of the *Shigella* species and ESBL encoding genes

Primers name		Sequence (5′→3′)	PCR product size (bp)	References
*invC*	F	TGCCCAGTTTCTTCATACGC	879	[17]
	R	GAAAGTAGCTCCCGAAATGC		
*wbgZ*	F	TCTGAATATGCCCTCTAC	430	[17]
	R	GACAGAGCCCGAAGAACCG		
*rfpB*	F	TCTCAATAATAGGGAACACAGC	537	[17]
	R	CATAAATCACCAGCAAGGTT		
*rfc*	F	TTTATGGCTTCTTTGTCG	211	[17]
	R	CTGCGTGATCCGACCATG		
*ipaH*	F	GTTCCTTGACCGCCTTTCCGATACCGTC	619	[18]
	R	GCCGGTCAGCCACCCTCTGAGAGTAC		
*bla*_*SHV*_	F	ATTTGTCGCTTCTTTACTCGC	1018	[19]
	R	TTTATGGCGTTACCTTTGACC		
*bla*_*CTX-M*_	F	ATGTGCAGYACCAGTAARGT	544	[19]
	R	TGGGTRAARTARGTSACCAGA		
*bla*_*TEM*_	F	ATAAAATTCTTGAAGACGAAA	1076	[19]
	R	GACAGTTACCAATGCTTAATC		
ERIC	F	ATGTAAGCTCCTGGGGATTCAC	Variable	[20]
	R	AAGTAAGTGACTGGGGTGAGCG		

### Phenotypic detection of ESBL-producing isolates

**Fig. 2:**
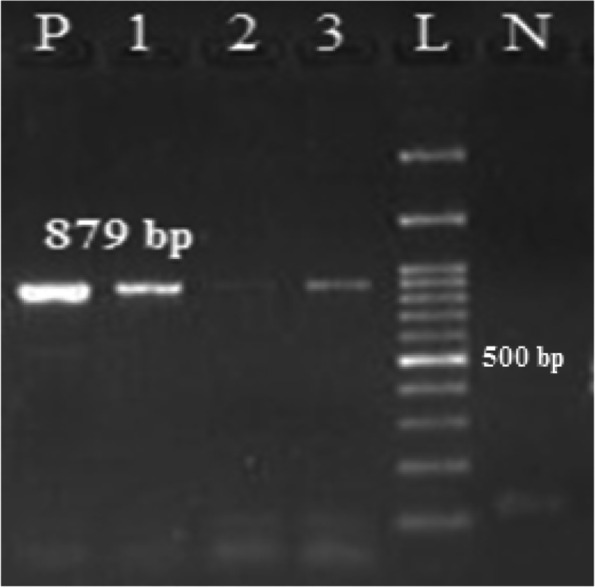
PCR product electrophoresis of invC gene. Lane L is related to DNA marker: 100 bp (*SMO321*, *Fermantas*); Lane P: positive control; Lane 1–3: positive samples; Lane N: negative control

ESBL production was determined according to the Clinical Laboratory Standards Institute (CLSI) guidelines [21]. Phenotypic detection of ESBL-producing isolates was carried out using ceftazidime (CAZ) and cefotaxime (CTX) disks (Becton Dickinson) and by the double-disk test on freshly prepared Mueller-Hinton agar (Fig. 2). Briefly, CAZ and CTX 30 μg disks, with and without clavulanic acid (CA) 10 μg disk, were used for testing. An inhibition zone ≥ 5 mm for CAZ or CTX tested in combination with CA versus its zone when tested alone was considered as ESBL-producing isolates. *Escherichia coli* ATCC 25922 was used as the standard strain.

### Molecular detection of ESBL encoding genes

A multiplex PCR assay targeting the main ESBL encoding genes including *bla*_*TEM*_, *bla*_*CTX-M*_, and *bla*_*SHV*_ was performed. The sequence of primers used for multiplex PCR assay is shown in Table 1. The multiplex PCR was performed at the final volume of 25 μl including 12 μl of 2 × Master Mix (SinaClon BioScience Company, Iran; Cat. no. PR901638) containing 3 mmol/l MgCl2, 0.4 mmol/l dNTPs, 1 × PCR buffer, 0.08 IU Taq DNA polymerase, 1 μl of 10 pmol of each forward and reverse primer, 3 μl of template DNA, and 8 μl of sterile distilled water. *Escherichia coli* ATCC 25922 was used as a standard strain.

### ERIC-PCR

The enterobacterial repetitive intergenic consensus-PCR (ERIC-PCR) was performed in a volume of 50 μl, containing 1 μl of bacterial genomic DNA (200–600 μl/ml), 3.7 μl of buffer TBE 10×, 1.5 μl of each dNTP (10 mM), 2.5 μl of (50 mM) 2Mgcl, 2 μl of each forward and reverse primer (90 pmol), and 1 μl of Taq DNA polymerase (5 units). Besides, the left volume was filled with 36.3 μl of PCR-grade water. The ERIC-PCR was conducted with the following conditions: initial denaturation of 95 °C for 4 min and 32 cycles of 95 °C for 60 s, 55 °C for 60 s, and 72 °C for 45 s followed by a final extension of 72 °C for 10 min. Primer sequences are presented in Table 1. The PCR products were stained with safe stain and electrophoresed on 2% agarose gel along with a 1-kb DNA ladder [20].

### Statistical analysis

The data were inputted and analyzed using the SPSS software ver. 23 (SPSS Inc., Chicago, IL, USA). A binary method and ward method were used for computing distance matrix and hierarchical clustering, respectively. The cophenetic value was determined as 0.647; this index measures the correlation of cophenetic (height) distance to the original distance in the data. To assess clustering tendency (the feasibility of the clustering analysis), Hopkins statistics was used, and its value was less than 0.5 (Hopkins statistic = 0.4), indicating that we could perform cluster analysis on the data set. All statistical analyses were performed in R software.

## Results

**Fig. 3:**
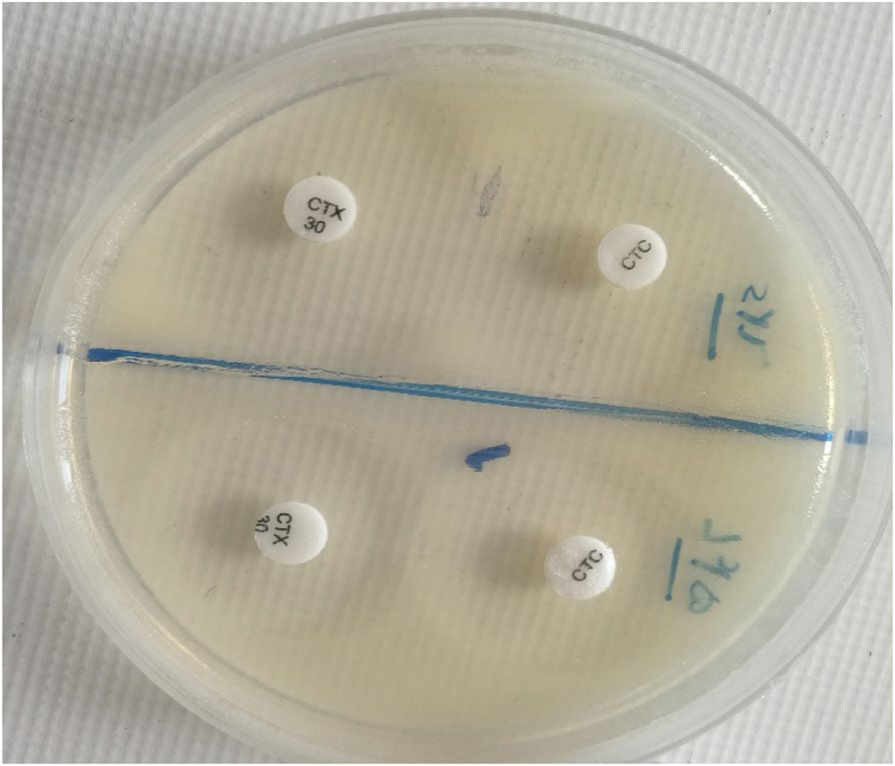
Phenotypic detection of ESBL-producing isolate using double disc test. The synergy between cefotaxime (CTX) and cefotaxime- clavulanate (CTC) is demonstrated

In the present study, a total of 1280 fecal samples were screened for *Shigella* genus. Conventional biochemical tests were employed for the identification and differentiation of *Shigella* spp. Based on biochemical tests, *Shigella* spp. was detected in 8.8% (*n* = 113/1280) of the fecal samples (Fig. 1). The prevalence of *Shigella* species among children with diarrhea is summarized in Table 2. Overall, *Shigella* species were isolated from 3.8% (*n* = 49/1280) of the samples using PCR assay (Fig. 3). The prevalence of *Shigella* species is given as follows: *S. sonnei* (*n* = 42 /49; 85.7%), *S. flexneri* (*n* = 5/49; 10.2%), and *S. dysenteriae* (*n* = 2/49; 4%). *S. boydii* was not detected in the fecal samples.

**Table 2:** The frequency of ESBL encoding genes in *Shigella* species

***Shigella*** species N (%)	ESBL	***bla***_***CTX***_	***bla***_***TEM***_	***bla***_***SHV***_
***S. flexneri*** ***N*** **= 5** (10.2%)	1 (20%)	3 (60%)	5 (100%)	0 (0%)
***S. dysenteriae*** ***N*** **= 2** (4.1%)	1 (50%)	1 (50%)	2 (100%)	0 (0%)
***S. sonnei*** ***N*** **= 42 (**85.7%**)**	3 (7.1%)	28 (66.7%)	23 (54.8%)	0 (0%)
***S. boydii*** ***N*** **= 0 (0%)**	-	-	-	-
**Total** ***N*** **= 49 (100%)**	5 (10.2%)	32 (65.3%)	30 (61.2%)	0 (0%)

ESBLs were produced by 5 out of 49 (10.2%) *Shigella* isolates including 3 *S. sonnei*, 1 *S. flexneri*, and 1 *S. dysenteriae*. The prevalence of ESBL-producing *Shigella* species is shown in Table 2.

**Fig. 4:**
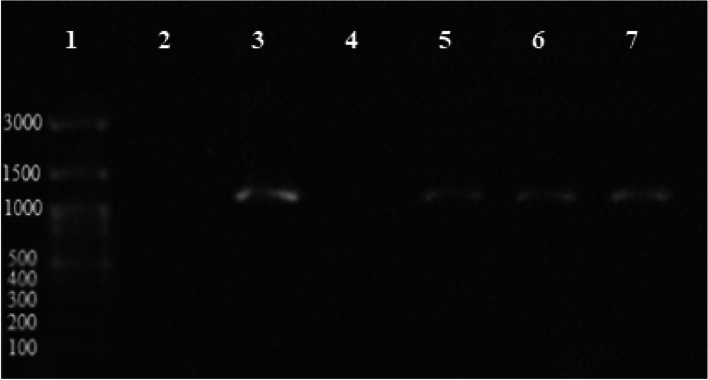
Illustration of *bla*_*TEM*_ PCR product on 1% agarose gel; lane 1, size marker (ladder 100 bp: *SMO321*, *Fermantas*); lane 2, negative control; lanes 3 and 5–7, positive *bla*_*TEM*_: 1076 bp samples

**Fig. 5:**
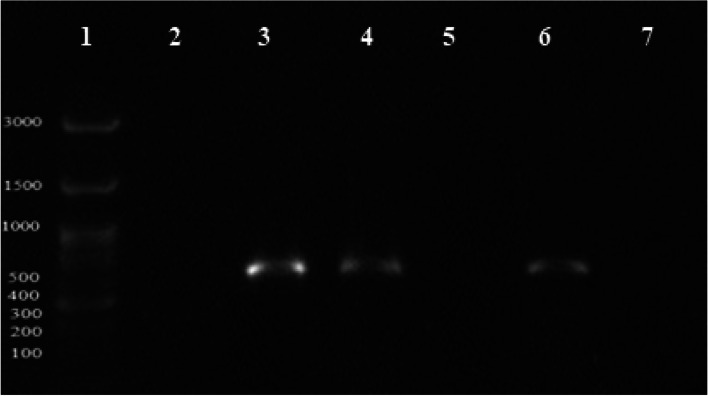
PCR product electrophoresis for the study of bla*CTX-M* gene on 1% agarose gel; lane 1, DNA marker (ladder 100 bp: *SMO321*, *Fermantas*); lane 2, negative control; lanes 3, 4, and 6, positive bla*CTX-M*: 544 bp

The ESBL encoding genes include *bla*_*CTX-M*_ and *bla*_*TEM*_ found in 65.3% and 61.2% of isolates, respectively (Figs. 4 and 5). *bla*_*SHV*_ gene was not detected in any isolates*.* The frequency of ESBL encoding genes among *Shigella* species is shown in Table 2.

### ERIC PCR analysis

**Fig. 6:**
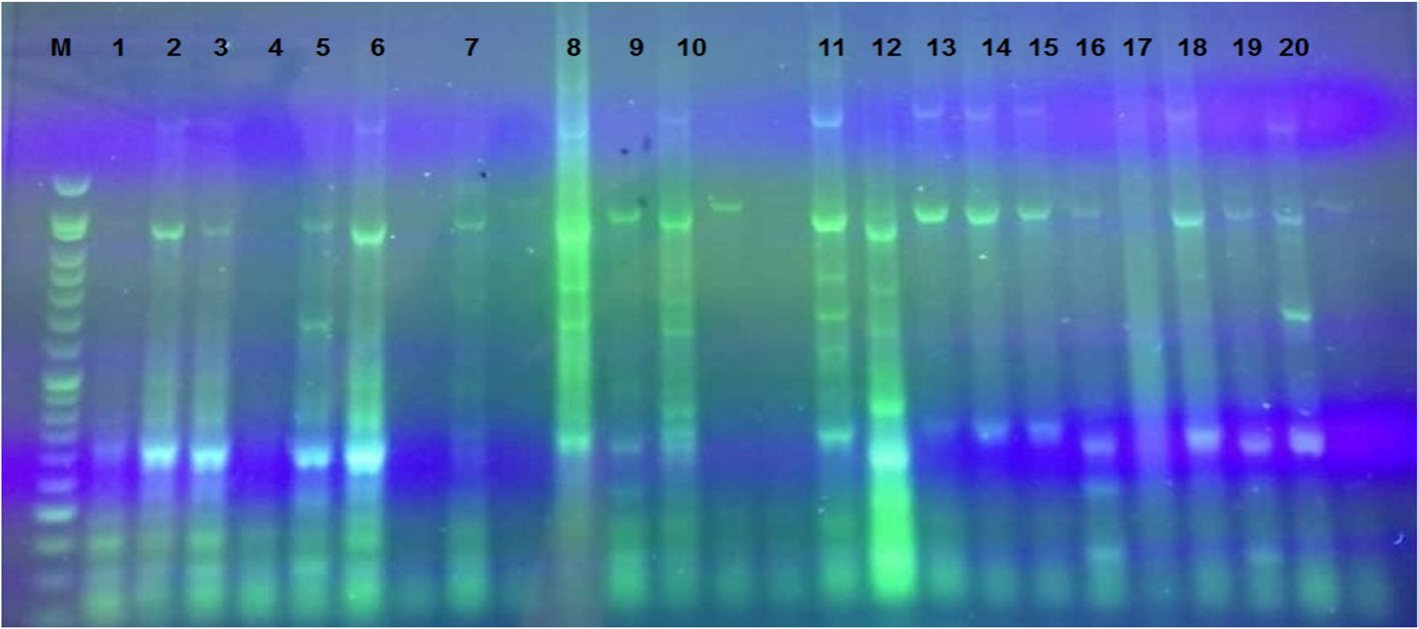
PCR product electrophoresis for the ERIC-PCR typing on 1% agarose gel; lane M: DNA marker (ladder100 bp: Cinnaclon, Iran)

**Fig. 7:**
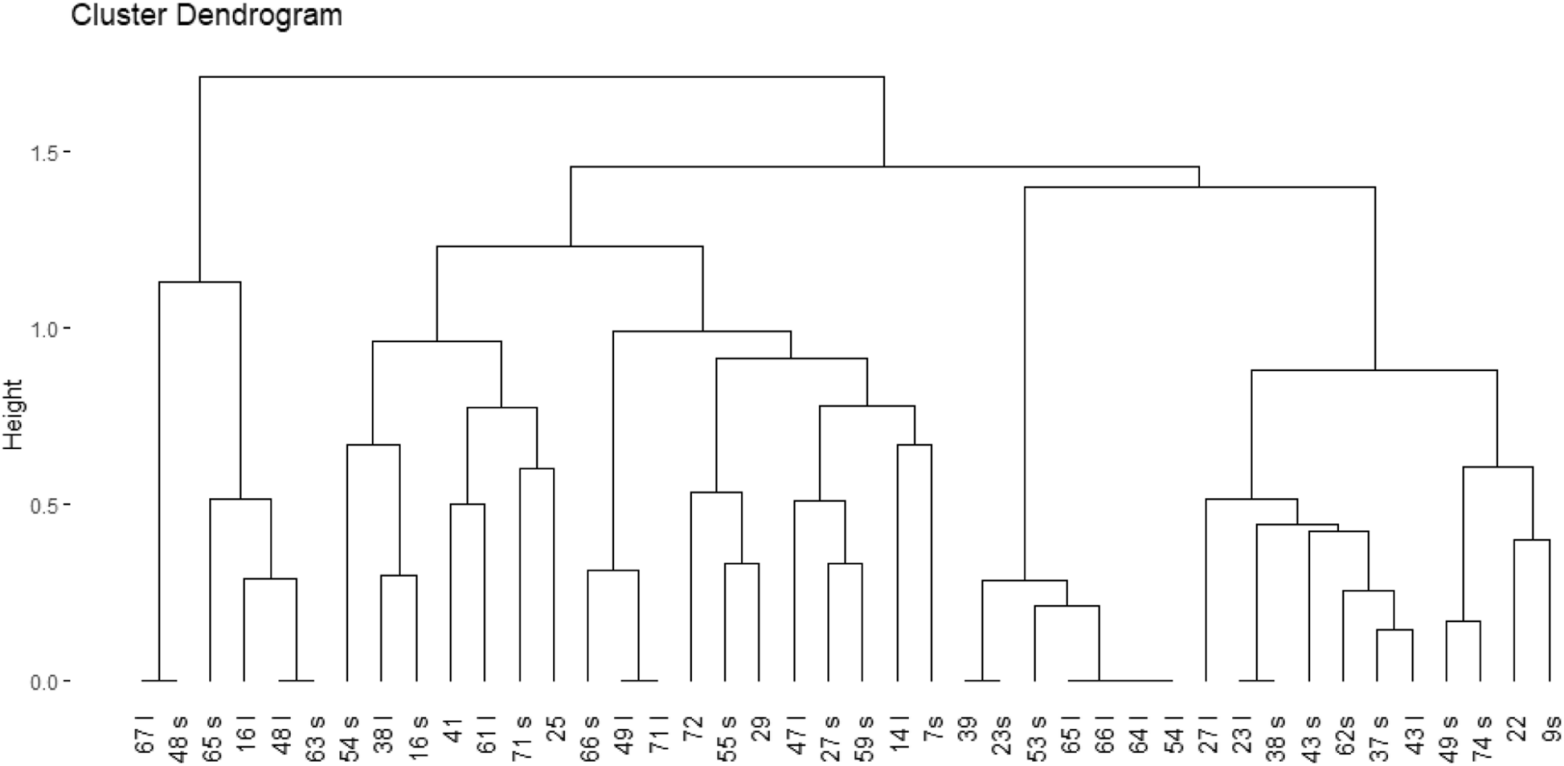
Dendrogram relating to isolated strains of *S. sonnei*

The genotyping profiles of 42 isolates of *S. sonnei* strains according to ERIC-PCR fingerprinting are shown in Figs. 6 and 7. All 42 *S. sonnei* under analysis produced 6–10 amplicons ranging from 100 to 1500 bp. The ERIC-PCR profiles allowed the differentiation of 42 *S. sonnei* strains into 6 clusters.

## Discussion

Among children in developing countries, despite the existence of effective treatment regimens, shigellosis continues to be the main public-health concern with an annual estimate of 163.2 million new cases and 1.1 million deaths [22]. In Iran, there are no specific guidelines to employ antibiotic therapy, and in most cases, physicians prescribe antibiotics without any stool cultures [23]. Therefore, performing epidemiological studies and gathering information regarding phenotypic and molecular mechanisms of antibiotic resistance to control infections and the development of local treatment guidelines are necessary.

In the present study, we surveyed the prevalence and molecular characterization of *Shigella* species harboring ESBL genes in patients with diarrhea from the northwest of Iran. This study showed a prevalence of 3.8% (49/1280) of shigellosis that was lower than the rates found in previously published studies conducted by Farajzadeh Sheikh et al. [6], Soltan Dallal et al. [24], Abbasi et al. [25], and Ranjbar et al. [20]. All of these studies were performed in Iran, and it was reported that the prevalence of *Shigella* spp. was 7.2%, 7.9%, 8.2%, and 9.4%, respectively.

However, the results of our study are in agreement with those of previous studies from Dhital et al. in Nepal [26], Aggarwal et al. in India [22], Jomehzadeh et al. from Iran [27], and Bakhshi et al. in Iran [28]. These studies found that the frequency of *Shigella* species in the patients with diarrhea was 2.1%, 1.9%, 5.9%, and 1.3%, respectively.

The detected variation in the prevalence of *Shigella* spp. could be due to the difference in the sample collection seasons, geography, specimen size, diversity of specimen type, study population, and applied detecting methods [27]. The result of the present study indicates that shigellosis is the main public health problem in the northwest of Iran. Therefore, it is critical to take several measures such as promoting awareness about the safety of water and food and improving health conditions.

Our finding revealed that *S. sonnei* had the highest frequency among *Shigella* species. In contrast, *S. boydii* was not detected in fecal samples. This result was in line with previous reports from Farajzadeh Sheikh et al. in Iran [29], Zhang et al. in China [30], Abbasi et al. in Iran [25], Bakhshi et al. in Iran [28], Ranjbar et al. in Iran [20], and Tajbakhsh et al. in Iran [31].

According to previous studies, it seems that in the southern regions of Iran, in the cities such as Shiraz, Ahvaz, and Kerman, *S. flexneri* is the dominant strain, while in northern regions of Iran, *S. sonnei* strain is prevalent [27, 32–36]. In contrast with our results, several different studies in various countries including Nepal [24], Ethiopia [37], Bulgaria [38], India [22], Iran [34], and Peru [39] have reported the *S. flexneri* as the leading cause of shigellosis.

In general, *Shigella* species can cause several diseases such as diarrheal in both developed and developing countries [40]. It has been reported that *S. flexneri* is the predominant serogroup in developing countries and is responsible for 44.5–80% of all *Shigella* infections such as *diarrhea*, while *S. sonnei* is a major cause of diarrheal in industrialized and developed countries [30].

In the present study, 10.2% were ESBL-producing strains by the double-disk method. Most ESBL-producing species were detected in *S. sonnei* which was following studies reported by Ranjbar [41]. In contrast, in a study performed by Aminshahidi et al., 54.5% of *S. flexneri* strain was ESBL positive [33].

Several different studies have surveyed the frequency of ESBL-producing *Shigella* strains. Results of a study performed by Li et al. from China revealed that ESBLs were produced by 18.1% of *Shigella* isolates, and most of the ESBL-producing species belonged to the *S. flexneri* isolates (19.5 %) [15]. In another study, Zhang et al. in China revealed that 10 *Shigella* isolates produced ESBLs including 8 *S. sonnei* isolates and 2 *S. flexneri* isolates [30].

Moreover, the prevalence of *ESBL-producing Shigella* isolates in the studies carried out by Aminshahidi et al. in Iran [33], Tau et al. in South Africa [42], Zamanlou et al. in Iran [43], Farajzadeh Sheikh et al. in Iran [29], Abbasi et al. in Iran [25], and Dhital et al. in Nepal [26] was > 50%, 0.3%, 54.2%, 43%, 52.6%, and 6.7%, respectively. In most studies, the highest prevalence of *S. sonnei* strains was ESBL producing.

The detected variation in the frequency of ESBL-producing *Shigella* species could be due to the differences in the applied detecting methods, variations in geographical location, and differences in sample size, sample type, and study participants [44]. Mobile genetic elements such as integrons, plasmids, and transposons can transmit the drug resistance genes among different bacteria and are responsible for antibiotic resistance in *Shigella* spp. [25]. In general, the main aim of antibiotic prescription in children with bloody and chronic diarrhea is the reduction in the duration of the disease. Because most *Shigella* infections are contagious and severe, appropriate antibiotic prescription and suitable treatment are essential [27].

Recently, the emergence of multidrug-resistant (MDR) *Shigella* species is increasing. The treatment of MDR strains is very difficult and is considered an alarming public health concern, worldwide [29].

In the present study, the *bla*_*CTX-M*_ with a frequency of 65.3% was the most predominant ESBL encoding gene followed by the *bla*_*TEM*_ gene with a frequency of 61.2% which was in line with previous reports by Andres et al. from Argentina [18], Vasilev et al. from Israel [19], Abbasi et al. from Iran [4], Farajzadeh Sheikh et al. from Iran [8], and Liu et al. from China [20].

*β-Lactamases are the enzymes encoded by several genes and were considered the main mechanisms of resistance to β-lactam antibiotics such as cephalosporins (*especially third-generation cephalosporins*) among gram-negative bacteria.* The high prevalence of ESBL-producing and β-lactamase genes leads to complicated treatment [33, 45].

Identification of ESBL among *Shigella* strains is an undeniable concern because it reduces antibiotic treatment options, and the spread of mobile resistance determinants will be a great threat to the treatment of invasive diseases in the future.

The present study has several limitations including the following: [1] the antibiotic susceptibility of *Shigella* species against different classes of antibiotics such as carbapenems was not determined, [2] the frequency of virulence genes was not determined, [3] the prevalence of other antibiotic resistance encoding genes such as genes encoding AmpC β*-*lactamase was not examined, and [4] the patients’ demographic data such as age, underlying disease, duration of hospitalization, and the outcome of treatment are not accessible; therefore, we were unable to perform deep analyses.

## Conclusion

The present study elucidated an update on the phenotypic and genotypic prevalence of ESBLs appearing among *Shigella* species circulating in the northwest of Iran. The high prevalence of *Shigella* species, especially *S. sonnei*, harbored ESBL genes in the present work; this is the main challenge for dysentery treatment, and it highlights the need for effective and regular monitoring of antibiotic usage among patients. Therefore, continued surveillance of the antimicrobial resistance profile and monitoring of the prevalence of *Shigella* species-producing ESBLs in Iran are urgently required. Epidemiological studies such as the present study provide valuable data on indigenous and resistant strains which help identify sources of infection, improve infection control systems, administrate effective drug treatment, and increase public health in the human community.

## Data Availability

All data generated or analyzed during this study are included in this published article.

## Abbreviations

DDT: Double-disk test
ESBL: Extended-spectrum β-lactamase
*XLD* agar: Xylose-*lysine deoxycholate* agar
HE: Hektoen enteric agar
*TSI*: *Triple sugar iron*
*SIM* medium: Sulfide indole motility medium
ODC: Ornithine decarboxylase
LIA: Lysine iron agar
CLSI: Clinical Laboratory Standards Institute
ERIC-PCR: Enterobacterial repetitive intergenic consensus-PCR
